# The Stroke-Induced Increase of Somatostatin-Expressing Neurons is Inhibited by Diabetes: A Potential Mechanism at the Basis of Impaired Stroke Recovery

**DOI:** 10.1007/s10571-020-00874-7

**Published:** 2020-05-23

**Authors:** Fausto Chiazza, Hiranya Pintana, Grazyna Lietzau, Thomas Nyström, Cesare Patrone, Vladimer Darsalia

**Affiliations:** 1 Department of Clinical Science and Education, Södersjukhuset, Internal Medicine, Karolinska Institutet, Stockholm, Sweden; 2 Department of Pharmaceutical Sciences, Università Degli Studi del Piemonte Orientale, Novara, Italy

**Keywords:** Somatostatin, Stroke, Type 2 diabetes, Neuroplasticity

## Abstract

Type 2 diabetes (T2D) hampers recovery after stroke, but the underling mechanisms are mostly unknown. In a recently published study (Pintana et al. in Clin Sci (Lond) 133(13):1367–1386, 2019), we showed that impaired recovery in T2D was associated with persistent atrophy of parvalbumin+ interneurons in the damaged striatum. In the current work, which is an extension of the abovementioned study, we investigated whether somatostatin (SOM)+ interneurons are also affected by T2D during the stroke recovery phase. C57Bl/6j mice were fed with high-fat diet or standard diet (SD) for 12 months and subjected to 30-min transient middle cerebral artery occlusion (tMCAO). SOM+ cell number/density in the striatum was assessed by immunohistochemistry 2 and 6 weeks after tMCAO in peri-infarct and infarct areas. This was possible by establishing a computer-based quantification method that compensates the post-stroke tissue deformation and the irregular cell distribution. SOM+ interneurons largely survived the stroke as seen at 2 weeks. Remarkably, 6 weeks after stroke, the number of SOM+ interneurons increased (*vs.* contralateral striatum) in SD-fed mice in both peri-infarct and infarct areas. However, this increase did not result from neurogenesis. T2D completely abolished this effect specifically in the in the infarct area. The results suggest that the up-regulation of SOM expression in the post-stroke phase could be related to neurological recovery and T2D could inhibit this process. We also present a new and precise method for cell counting in the stroke-damaged striatum that allows to reveal accurate, area-related effects of stroke on cell number.

**Electronic supplementary material:**

The online version of this article (10.1007/s10571-020-00874-7) contains supplementary material, which is available to authorized users.

## Introduction

Type 2 Diabetes (T2D), a major risk factor for stroke, dramatically hampers neurological recovery in the surviving patients (Jorgensen et al. 1994; Baird et al. 2002; Megherbi et al. 2003) and is a strong predictor of persistent dependency on assistance in activities of daily living (ADL) (Ullberg et al. 2015). There are several possible mechanisms wherein diabetes leads to stroke. These include vascular endothelial dysfunction, increased early-age arterial stiffness, systemic inflammation and thickening of the capillary basal membrane (Chen et al. 2016).

Impaired neurological recovery in T2D cannot be explained by only more severe brain damage after stroke (Sweetnam et al. 2012; Tulsulkar et al. 2016; Dhungana et al. 2013) and several mechanisms including impaired vascular restoration (Ergul et al. 2015), increased inflammation (Dhungana et al. 2013) and impaired stroke-induced neurogenesis (Zhang et al. 2016; Pintana et al. 2019) have been proposed. Neuroplasticity, i.e. the ability of the nervous system to respond to stimuli by reorganizing its structure, function and connections, is also considered an important mechanism involved in post-stroke recovery (Alia et al. 2017; Mang et al. 2013) and different types of neurons play key roles in this process (Greifzu et al. 2014; Quattromani et al. 2018; Wieloch and Nikolich 2006). Interestingly, T2D has been reported to diminish cortical plasticity (Sweetnam et al. 2012) in rodents and importantly, in patients (Huynh et al. 2017). Moreover, we recently showed that impaired stroke recovery in T2D may also be associated with increased atrophy of striatal GABA-ergic parvalbumin (PV)+ interneurons (Pintana et al. 2019), suggesting diminished neuroplasticity in striatum.

Apart from PV+ interneurons, other interneurons could be involved in stroke recovery and among them, somatostatin+ (SOM+) neurons are plausible candidates. SOM is a small 14 amino-acid-long peptide present in many regions of the central and peripheral nervous system (Patel 1999). In the central nervous system (CNS), positive correlation between the amount of expressed somatostatin and increased performance in hippocampus-dependent learning tasks has been observed (Liguz-Lecznar et al. 2016; Nilsson et al. 1993). Additionally, SOM+ interneurons were demonstrated to be involved in driving neuroplasticity and contributing to the reshaping of neural networks in three characterized neuroplasticity models (ocular dominance changes, classical fear conditioning and experience-dependent barrel cortex plasticity) (Tang et al. 2014). SOM+ cells largely co-stain with Neuropeptide Y (NPY) (Rushlow et al. 1995), a 36-amino acid residue long peptide implicated in regulations of important biological and pathophysiological functions such as neuroendocrine secretions, feeding behavior, circadian rhythms, seizures, neuronal excitability, neuroplasticity, and memory (Gotzsche and Woldbye 2016). Interestingly, in models of brain ischemia, there is a selective sparing of SOM/NPY expressing interneurons in striatum as demonstrated in a gerbil and in a rat model of global forebrain ischemia at different time points after stroke (4 days and 2–28 days respectively) (Uemura et al. 1990; Larsson et al. 2001). Moreover, this selective interneurons sparing was confirmed in a more complex animal model of ischemia, as showed by Mallard et al. in the brain of sheep fetuses that underwent to repeated hypoxia (Mallard et al. 1995). Specifically, in the striatum the number of these neurons is usually reduced shortly after stroke (4 h/1 day) followed by normalization to baseline-like values in 3/7 days (Grimaldi et al. 1990), likely due to down- and up-regulation of somatostatin expression. Furthermore, this effect seems to be influenced by the stroke-damaged brain area in which SOM/NPY cell number is evaluated, resulting in a delay in the cortex (40 days for complete recovery) and complete absence in the hippocampus (no recovery after stroke) (Grimaldi et al. 1990). This regulation pattern suggests that this interneuron type may play a specific role in post-stroke recovery. However, the potential role of these cells to affect stroke recovery is undetermined. Moreover, whether T2D affects SOM+ interneuron survival after stroke has not been previously investigated.

To determine whether SOM+ interneurons are affected during the recovery phase after stroke, it is necessary to evaluate the density of SOM+ cells in the striatum discriminating between areas more (infarct area) or less (peri-infarct area) affected by the ischemic injury. As these interneurons are not uniformly distributed in striatum (Takagi et al. 1983) (i.e. they concentrate mainly adjacent to the lateral ventricle, which is the area usually less affected in focal stroke models), simply comparing the number of SOM+ cells between contralateral and ipsilateral hemispheres is not precise. Moreover, the deformation of tissue after stroke should be considered in this kind of quantitative evaluation.

The aim of this study was to determine whether T2D affects the survival of striatal SOM+ interneurons in the post-stroke recovery phase in the mouse at 2 and 6 weeks after stroke. To precisely quantify SOM+ interneurons in peri-infarct and infarct areas, we set up a new computerized methodology that takes advantage of the complementary use of microscopy and an image-editing software. This study represents the extension of a previous study carried out by our group (Pintana et al. 2019) where we employed an experimental design of clinical relevance in which T2D was induced by 12 months feeding with high-fat diet (HFD). In this setting, we showed that the neurological impairment after stroke in HFD-fed mice was similar to healthy, standard diet (SD)-fed mice at 2 weeks after tMCAO (Pintana et al. 2019). However, while SD-fed mice recovered at 6 weeks after stroke, neurological function remained significantly impaired in HFD-fed mice (Pintana et al. 2019).

## Materials and Methods

### Animal Models and Experimental Design

All experiments were conducted in accordance with the Guidelines for Care and Use of Laboratory Animals published by US National Institute of Health (Eighth edition 2011) and approved by the regional ethics committee (Stockholm Södra Djurförsöketiska Nämnd) for animal experimentation (application No S7–13). This study represents the extension of a previous study (Pintana et al. 2019). Therefore, the material from the same mice cohort was used to obtain the data showed in this paper. This is in line with the guideline principles at our institution, with the aim to improve the ethical use of animals in testing according to the 3 R principle (Balls 2009).

Briefly, thirty-nine, male 8-week-old C57/BL6j mice (Charles River Laboratories, Germany) were used. The mice were randomized to two groups: fed with standard laboratory diet (SD) (*n* = 22) or high-fat diet (HFD: 60% energy from fat) (*n* = 17) for 12 months. All mice were housed in environmentally controlled conditions (25 ± 0.5 °C, 12/12 h light/dark cycle with ad libitum access to food and water). T2D and obesity were defined as fasting blood glucose over 7 mmol/L and weight gain over 15% respectively (the data are presented in the Supplementary material of Pintana et al. 2019). After 12 months of the diet regimen, mice were subjected to experimental stroke (SD, *n* = 18; HFD, *n* = 13) or sham surgery (SD, *n* = 4 HFD, *n* = 4). Then, mice were divided into two studies:

### Study 1

21 mice subjected to tMCAO (SD, *n* = 13; HFD, *n* = 8) plus 8 shams mice (SD, *n* = 4 HFD, *n* = 4) were sacrificed for histological assessment after 6 weeks.

### Study 2

10 mice subjected to tMCAO (SD, *n* = 5, HFD, *n* = 5) were killed after 2 weeks for additional histological analysis at this earlier time point.

To mark newly born cells, all mice received daily intraperitoneal injections of the thymidine analog bromodeoxyuridine (BrdU; 50 mg/kg of BW) for 2 weeks following tMCAO.

The results about metabolic parameters, stroke brain damage and functional recovery after tMCAO have been recently published (Pintana et al. 2019) and are summarized in the Results section.

### Transient Middle Cerebral Artery Occlusion (tMCAO)

tMCAO was used to model stroke. tMCAO was induced by intraluminal filament technique (Hara et al. 1996). To induce striatal infarct with minimal cortical and hippocampal damage, middle cerebral artery (MCA) was occluded for 30 min. Briefly, mice were anesthetized by 3% isoflurane, then maintained by 1.5% isoflurane through a snout-mask throughout the surgery. Body temperature was maintained at 37–38 °C using a heated pad. Through midline incision, left common, external and internal carotid arteries were exposed. Through an incision in an external carotid artery, a 15 mm long, 7–0 silicone-coated monofilament (total diameter 0.17–0.18 mm) was inserted into the internal carotid artery until it could not be advanced any further and at least 8–10 mm of filament length has passed the carotid bifurcation thus blocking the origin of the MCA. Then, wounds were temporarily closed, and mice were allowed to wake up. After 25 min mice were re-anesthetized, wound reopened and the occluding filament removed (total tMCAO time 30 min). Additionally, the success of the stroke-induction was evaluated by scoring the severity of neurological deficits 1 h after reperfusion and the next day after tMCAO surgery using a 3-point system: 1 point for circling towards the paretic side, 1 point for right forelimb sensory deficit (left hanging from the table edge) and 1 point for hind limb sensory deficit. All animals used in the study reached a minimum of 2 points (data not shown).

### Immunohistochemistry

Brain preparation was performed as described previously (Chiazza et al. 2018; Darsalia et al. 2016; Lietzau et al. 2018, 2020). Mice were deeply anesthetized and transcardially perfused with phosphate-buffered saline (PBS) followed by 4% ice-cold paraformaldehyde, then the brains were removed and after overnight post-fixation submersed in PBS containing 20% sucrose until they sank. The brains were cut in 30-μm-thick coronal sections using a sliding microtome. Immunofluorescence staining was performed using free-floating method. Following primary antibodies were used; rabbit anti-somatostatin (SOM) (1:1,500 dilution; #20067; Immunostar); rat anti-somatostatin (SOM) (1:500; #sc-47706; Santa Cruz); rabbit anti-neuropeptide Y (NPY) (1:1,000 dilution; #22,940; Immunostar); mouse anti dopamine- and cAMP-regulated neuronal phosphoprotein-32 (DARPP-32) (1:250 dilution; #sc-271111; Santa Cruz); rabbit anti Calretinin (Cr) (1:300 dilution; #VP-RM11; Vector laboratory); rabbit anti Choline acetyltransferase (ChAT) (1:200 dilution; #AB143; Millipore) mouse anti-NeuN (1:200 dilution; #MAB377; Millipore); goat anti Ionized calcium-binding adaptor molecule 1 (Iba-1) (1:1,000 dilution; #ab5076; Abcam); rat anti-Bromodeoxyuridine (BrdU) (1:500 dilution; #ab6326; Abcam); mouse anti-parvalbumin (PV) (1:1,500 dilution; #P3088; Sigma); rabbit anti Oligodendrocyte transcription factor (Olig-2) (1:500 dilution; #AB9610; Millipore); rabbit anti-Glial fibrillary acidic protein (GFAP) (1:2,000 dilution; # Z0334; DAKO); rabbit anti-S100beta (1:3000 dilution; # ab41548; Abcam). Sections were incubated with primary antibodies overnight at 4 °C in a phosphate buffer containing 3% appropriate serum and 0.25% Triton X-100. Primary antibodies were detected by Alexa 488-or Alexa 594-conjugated (Vector laboratory), Cyanine3-conjugated (Thermo Fisher scientific) or biotin-conjugated (Vector Laboratories, Sweden) secondary antibodies (1:200 dilution). Sections were incubated with secondary antibodies for 2 h at room temperature (approximately 21 °C) in phosphate buffer containing 3% of the appropriate serum and 0.25% Triton X-100. For chromogenic visualization, avidin–biotin complex (ABC kit, Vector Laboratories, Sweden) and diaminobenzidine (DAB; Sigma-Aldrich, US) were used. For antigen retrieval 1 mM EDTA for 30 min at 69 °C was applied for double staining, or 1 M HCl for 20 min at 64 °C (for double staining between rat anti BrdU and rabbit anti SOM).

### Quantitative Microscopy

The Olympus BX51 epi-fluorescent/light microscope (Olympus) connected with computerized setup for stereology (NewCast Software, Visiopharm) were used for cell counting. All quantifications were performed by an investigator blinded to group identities. Two counting methods were used and compared.

*Method 1* three consecutive brain sections spaced at 300 μm containing striatum (from Bregma 1 to 0.5 mm) were used. The first section was chosen based on an anatomical location along the rostra-caudal axis (approximately 1 mm from Bregma). The second and the third sections were 300 and 600 μm caudal from the first section respectively. Immuno-positive cells were counted using the NewCast software (VisioPharm, Denmark). Briefly, striatum on 3 coronal sections (described above) was delineated and the counting frame was systematically moved at preset intervals starting from random position, so that the representative fraction of the region of interest was sampled. The total cell number from three sections was estimated using the following formula: Total number of immuno-reactive cells = Counted number × (Step area/Counting frame area) (Chiazza et al. 2018; Pintana et al. 2019).

*Method 2* all positive cells were counted in striatum in one section (≈ 0.5 mm from Bregma).

In both counting methods the mean of the contralateral immuno-positive cell number was calculated, and the ipsilateral cell number was normalized to the contralateral mean. In this way we obtained the mean of contralateral cell number set to 100% in both methods, and ipsilateral cell number expressed as percentage change compared to contralateral. The two counting methods produced identical results (Fig. S1); therefore, all quantifications were performed in a single brain section from each animal by using Method 2.

### Cell Density Evaluation

To evaluate the differences in SOM+ cells in the infarct and peri-infarct areas of the ipsilateral hemisphere, cell density per mm^2^ was calculated. The density of SOM+ cells was calculated by dividing the number of SOM+ cells by the area (mm^2^) in which these cells were quantified.

For the evaluation of the density of SOM+ cells in the ipsilateral hemisphere, the post-stroke deformation of the striatum was considered. From our previous studies (Fig. S2) there is no deformation at all in the Peri-Infarct area of the striatum after stroke, but only in the area where there is an infarct. Thus, to extrapolate the “deformation coefficient” of the infarct area, this formula was used:

$${\alpha }_{\text{s}}=\frac{C-P}{I},$$

*α*_s_ is the deformation coefficient, *C* contralateral striatal area, *P* ipsilateral striatal peri-infarct area, *I* ipsilateral striatal infarct area.

The deformation coefficient was used to correct the density of SOM+ cells in the ipsilateral infarct area using this formula:

$${\delta }_{\text{c}}=\frac{{\delta }_{\text{o}}}{{\alpha }_{\text{s}}},$$

*δ*_c_ is the real cell density (corrected), *δ*_o_ is the cell density obtained, *α*_s_ is the deformation coefficient.

This formula was used only to correct the density in ipsilateral infarct area, being that, as already stated, peri-infarct area is not affected by the deformation (shrinking in this case).

**Fig. 1:**
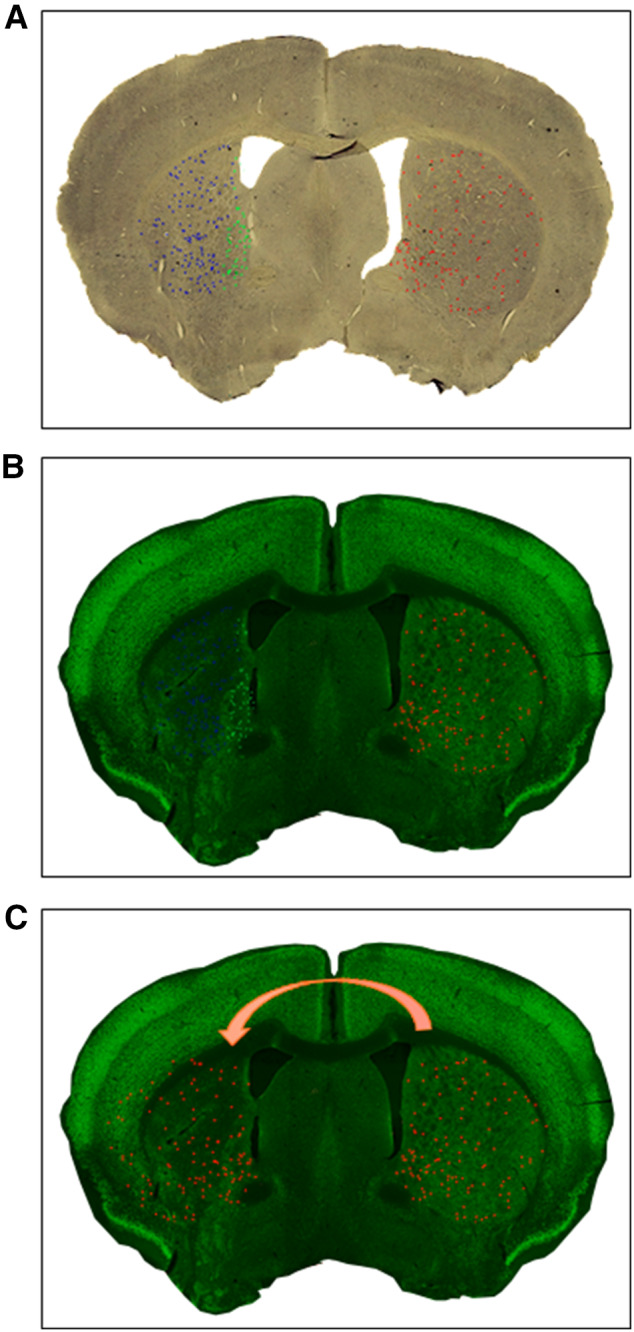
Representation of the protocol for a new method for the evaluation of Somatostatin (SOM)+ cell density. A high-resolution image of the brain section from each animal (≈ 0.5 mm from Bregma) stained for SOM was superimposed over a high-resolution, image of a similar brain section from the same animal stained for NeuN. On these two images, was further superimposed a diagram of the position of the SOM+ cells from the contralateral and ipsilateral striata (**a**, **b**). By using this diagram over-posed to NeuN stained section, SOM+ cells in the ipsilateral were identified and divided in peri-infarct and infarct area (green and blue dots respectively). Then the diagram of the Contralateral SOM+ cells was mirrored and over-imposed to the ipsilateral NeuN stained striatum and cells in the corresponding peri-infarct area were counted (**c**). Finally, the number of SOM+ cells in the contralateral infarct corresponding area was calculated by subtracting the number of SOM+ cells in the contralateral peri-infarct corresponding area from the total number of SOM+ cells in the whole contralateral

From literature (Takagi et al. 1983) and from data collected for this study, SOM+ cells distribution in striatum is not uniform. While this non-uniformity is negligible under normal conditions, it gains great importance in the case of focal striatal stroke, as striatal SOM+ cells are more concentrated in the area adjacent to the lateral ventricle, and this area is usually the least affected by the tMCAO. This feature was considered in the SOM+ cells density comparison of contralateral vs. ipsilateral. Thus, the density of the SOM+ cells in the ipsilateral peri-infarct area was compared to the density in the anatomically corresponding contralateral area, and not to the SOM+ cells density in the whole contralateral striatum. Similarly, the density of the SOM+ cells in the Ipsilateral infarct area (corrected as described above) was compared to the anatomically corresponding contralateral area. To correctly delineate the corresponding peri-infarct or infarct area in the contralateral hemisphere, an image-editing software was used (Adobe Photoshop CC 2019). Briefly, a high-resolution image of the brain section from each animal (≈ 0.5 mm from Bregma) stained for SOM was superimposed over a high-resolution, image of a similar brain section from the same animal stained for NeuN. On these two images, was further superimposed a diagram of the position of the SOM+ cells from the contralateral and ipsilateral striata (Fig. 1a, b). By using this diagram over-posed to NeuN stained section, SOM+ cells in the ipsilateral were identified and divided in peri-infarct and infarct area (green and blue dots respectively). Then the diagram of the Contralateral SOM+ cells was mirrored and over-imposed to the ipsilateral NeuN stained striatum and cells in the corresponding peri-infarct area were counted (Fig. 1c). Finally, the number of SOM+ cells in the contralateral infarct corresponding area was calculated by subtracting the number of SOM+ cells in the contralateral peri-infarct corresponding area from the total number of SOM+ cells in the whole contralateral.

All procedures were performed by experimenter blinded to experimental groups.

### Statistical Analysis

The significance of the differences between 2 groups was calculated using the unpaired *t* test with Welch’s correction. The comparisons between three groups were performed using the one-way ANOVA test with Bonferroni multiple comparisons. Data are expressed as mean ± SEM. *P*-value less than 0.05 was considered statistically significant. All data were analyzed by using GraphPad Prism 7.

## Results

### Effects of T2D on Metabolic Parameters, Stroke Volume and Functional Recovery After tMCAO

As stated above, this study is based on the reuse of the material from a previously published project from our group (Pintana et al. 2019). The metabolic results as well as the stroke volume and functional recovery data are summarized below. Briefly, 12 months of HFD induced an impaired metabolic profile, determined by a significant increase in body weight, increased fasting glycemia and impaired glucose tolerance (Pintana et al. 2019). The ischemic brain damage was mainly localized in dorsolateral striatum. No significant differences between SD- and HFD-fed mice were detected in stroke volume or in upper-limb grip strength between SD-fed mice and HFD-fed mice before stroke (Pintana et al. 2019). Stroke significantly decreased upper-limb grip strength (before stroke vs. 1 week) in both SD and HFD-fed mice. However, at 6 weeks after stroke, SD-fed mice significantly recovered upper-limb grip strength (1 vs. 6 weeks, *P* < 0.0001), while HFD-fed mice did not and remained significantly different from before stroke and HFD sham (Pintana et al. 2019).

### Stroke Increases SOM+ Interneurons in the Whole Striatum 6 Weeks After Stroke; An Effect that is Partially Hampered by T2D

**Fig. 2:**
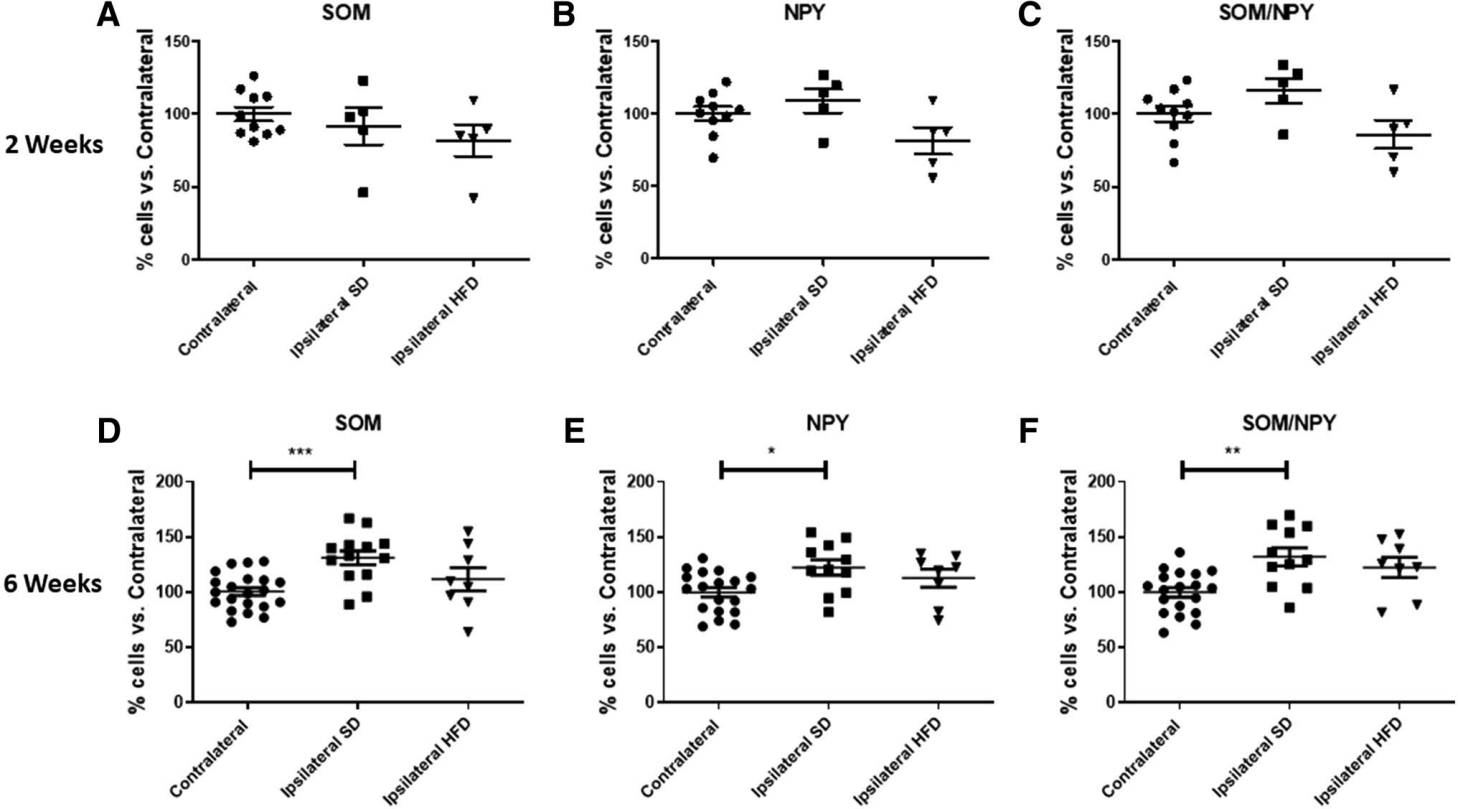
Effect of Type 2 diabetes (T2D) on stroke-induced somatostatin (SOM)+ or neuropeptide Y (NPY)+ single staining and SOM+/NPY+ double staining cell number 2 and 6 weeks after transient Middle Cerebral Artery Occlusion (tMCAO). The percentage of the SOM+ cell, NPY+ cell and SOM+/NPY+ cell number after normalization for the mean value of respective contralateral at 2 weeks (**a**, **b** and **c**) and 6 weeks after stroke (**d**, **e** and **f**), respectively in mice fed a standard diet (SD) or a High Fat Diet (HFD). One-way ANOVA test with Bonferroni post-test, means ± SEM. *P < 0.05; **P < 0.01; ***P < 0.001

Two weeks after stroke, no differences were observed in the number of SOM+, NPY+ and SOM+/NPY+ co-stained cells in ipsilateral versus contralateral striatum in both animals fed a SD or an HFD (Fig. 2a–c). These results confirm the selective sparing of this interneuron subtype after stroke (Grimaldi et al. 1990; Mallard et al. 1995; Larsson et al. 2001).

Six weeks after stroke, SOM+ cells number was significantly increased in ipsilateral striatum of SD-fed mice compared to contralateral (*P* < 0.01, Fig. 2d). This stroke-dependent effect did not occur in HFD-fed mice (*P* = 0.35, Fig. 2d). Similar results were obtained when assessing NPY+ cells or SOM+/NPY+ co-staining cells (Fig. 2e, f).

This increase of SOM+ in SD-fed mice appears to occur in the ischemia injured hemisphere, and not because of a change of SOM+ cells in contralateral striatum, as no differences were detected in cell number between sham brains and contralateral hemispheres (Fig. S2).

Overall these results confirm the remarkable resistance of SOM+ and NPY+ cells up-to 2 weeks after stroke. Importantly, our results show that stroke increased SOM+ and NPY+ cell number 6 weeks after stroke in SD mice while T2D prevented this stroke-dependent effect.

### Type 2 Diabetes Prevents the Stroke-Induced Increase of SOM+ Interneurons in the Infarct Stroke Area Without Affecting the Peri-infarct Area

**Fig. 3:**
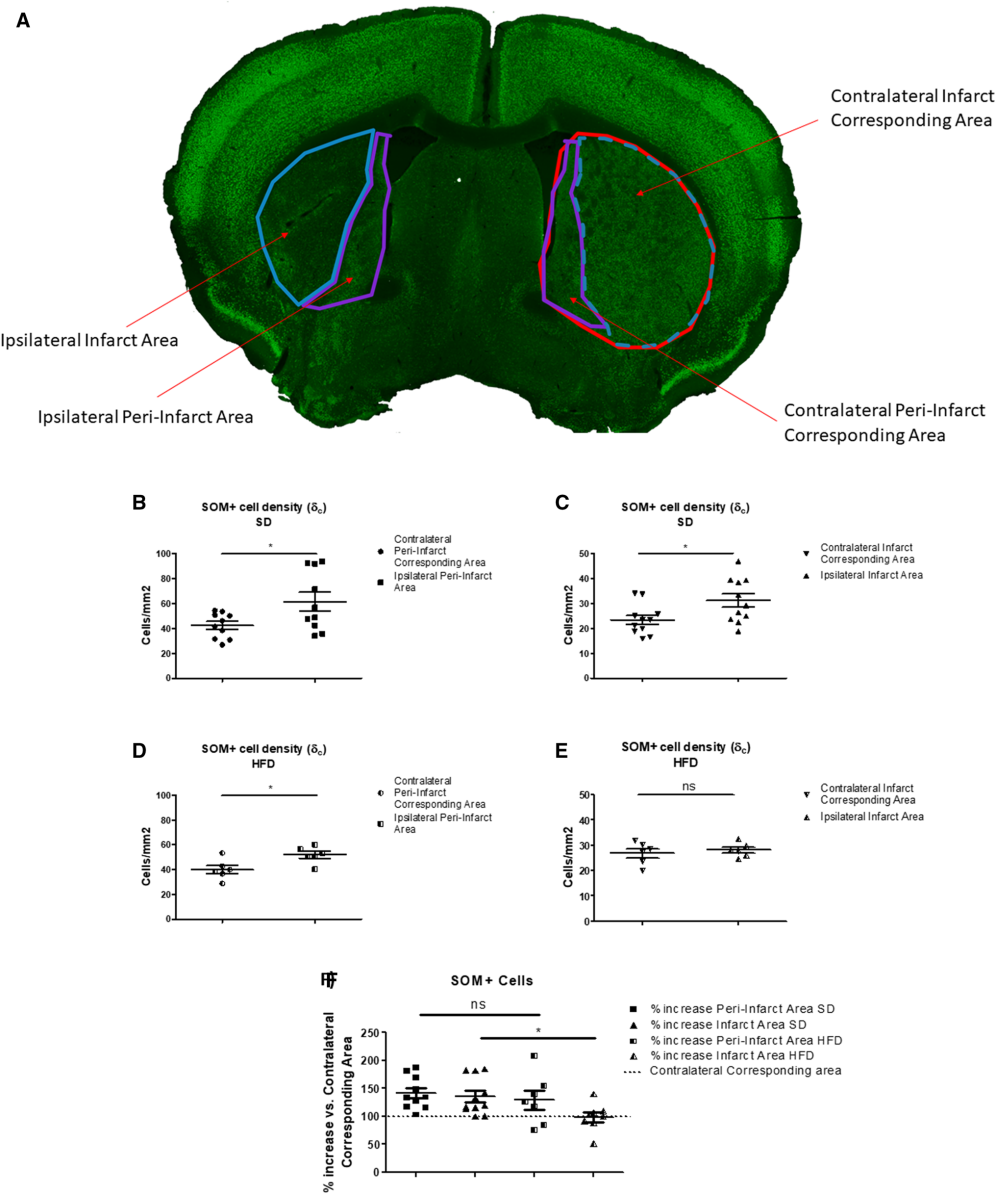
Effects of T2D on Type 2 diabetes (T2D) on stroke-induced Somatostatin (SOM)+ cells density (*δ*_c_) increase 6 weeks after stroke discriminating between peri-infarct and infarct area. Representation of the areas in which striatum was divided for cell density analysis (*δ*_c_) (**a**). SOM+ cell density (*δ*_c_) in peri-infarct area in ipsilateral striatum compared to contralateral peri-infarct corresponding area in Standard Diet (SD) fed mice 6 weeks after stroke (**b**). SOM+ cell density (*δ*_c_) in infarct area in ipsilateral striatum compared to contralateral infarct corresponding area (adjusted for infarct volume shrinking) in SD-fed mice 6 weeks after stroke (**c**). SOM+ cell density (*δ*_c_) in peri-infarct area in ipsilateral striatum compared to contralateral peri-infarct corresponding area in High Fat Diet (HFD) fed mice 6 weeks after stroke (**d**). SOM+ cell density (*δ*_c_) in infarct area in ipsilateral striatum compared to contralateral infarct corresponding area (adjusted for infarct volume shrinking) in HFD-fed mice 6 weeks after stroke (**e**). Stroke-induced SOM+ cell increase in peri-infarct and infarct areas in ipsilateral striatum compared to contralateral corresponding area (dotted line) in SD and HFD-fed mice 6 weeks after stroke (**f**). Unpaired *t* test with Welch’s correction, means ± SEM *P < 0.05

The quantitative analysis of the whole ipsilateral SOM+ or NPY+ cells number was not sufficiently sensitive to reveal a statistical difference between ipsilateral SD and ipsilateral HFD (Fig. 2d–f). Therefore, to further deepen our investigation, we set up a new methodological approach to calculate cell density at 6 weeks after stroke to separately assess the stroke-dependent variation in SOM+ cell number in peri-infarct and infarct areas (Fig. 3a). We also adjusted our quantifications based on the stroke-induced striatum deformation (Fig. 3a and “Materials and Methods” section for further details). Due to the similar results in the quantifications of SOM+, NPY+ and SOM+/NPY+ interneurons (Fig. 2d–f) and considering that almost 100% of the NPY+ cells in striatum co-stain with SOM cells (Rushlow et al. 1995), we focused the cell density (*δ*_c_) quantifications solely in SOM+ cells (see “Materials and Methods” section).

The results show that 6 weeks after stroke SD-fed mice increased approximately 40% the adjusted SOM+ *δ*_c_ in ipsilateral peri-infarct (*P* < 0.05; Fig. 3b, f) and infarct (*P* < 0.05; Fig. 3c, f) areas if compared to their corresponding contralateral areas. T2D showed a similar pattern in the *δ*_c_ of SOM+ interneurons in the peri-infarct area (*P* < 0.05; Fig. 3d, f). However, T2D entirely abolished the increase of SOM+ *δ*_c_ in the infarct area (*P* = 0.6; Fig. 3e, f).

Overall these results indicate a significant effect of T2D to block the increase of SOM+ interneurons in the stroke infarct area 6 weeks after stroke. This effect could not be obtained by quantifying SOM+ cells in the whole striatum, and without considering the stroke-induced striatal deformation effect, thus demonstrating the increased sensibility of our newly established quantitative method.

### The Increased Number of SOM+ Interneurons After Stroke Does Not Result From Neurogenesis or Increased SOM Expression in Other Neuronal or Glial Cell Populations

To investigate if the increased number of SOM+ interneurons resulted from an up-regulation of this marker in SOM− cells, we performed co-staining of SOM with several neuronal and glial markers in ipsilateral striatum of mice fed a SD 6 weeks after stroke.

**Fig. 4:**
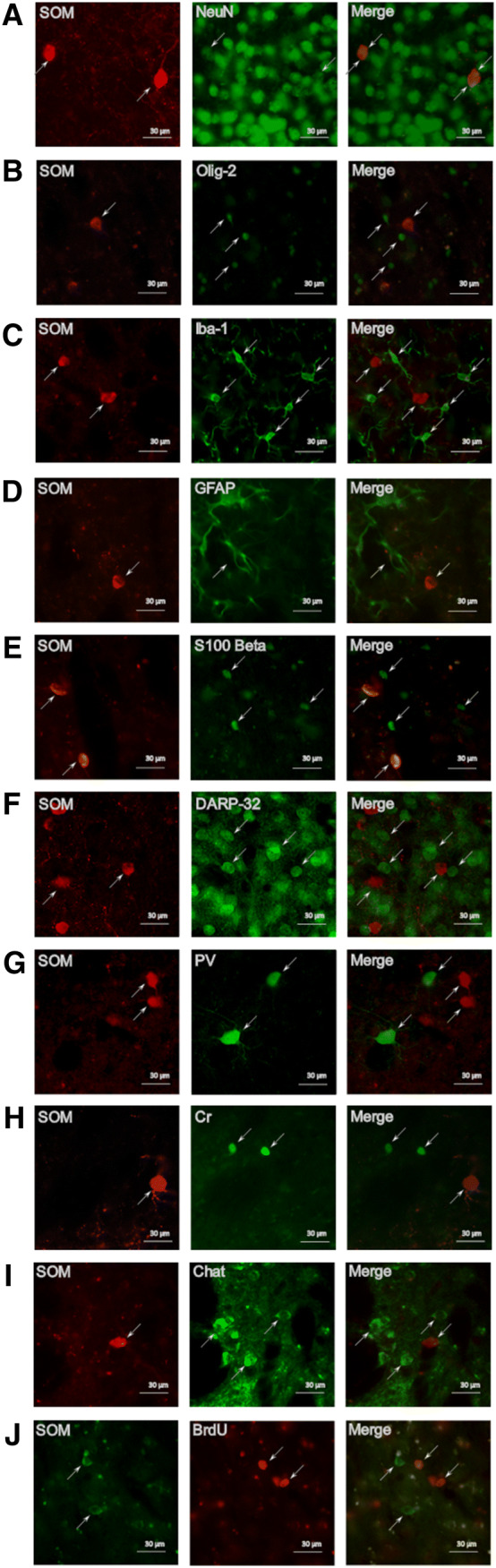
Characterization of SOM+ cells. Representative images from ipsilateral striatum of SOM+ cells co-staining with NeuN (**a**), Oligodendrocyte transcription factor (Olig-2) (**b**), Ionized calcium-binding adaptor molecule 1 (Iba-1) (**c**), Glial fibrillary acidic protein (GFAP) (**d**), S100Beta (**e**); dopamine- and cAMP-regulated neuronal phosphoprotein-32 (DARPP-32) (**f**), parvalbumin (PV) (**g**), Calretinin (Cr) (**h**), choline acetyltransferase (ChAT) (**i**), bromodeoxyuridine (BrdU) (**j**), at 6 weeks after stroke

We found a 100% overlap between SOM and NeuN (Fig. 4a), a ubiquitous marker of mature neurons, confirming the neuronal nature of SOM+ cells. We further confirmed this result as no co-staining was detected between SOM and Olig-2 (a marker of the oligodendrocytes lineage) (Fig. 4b), Iba-1 (a marker of microglia) (Fig. 4c) and GFAP or S100 Beta (2 markers of astroglia) (Fig. 4d, e). Since, SOM expression was also absent in spiny neurons, as assessed by co-staining between SOM and the spiny neuronal marker DARPP-32 (Fig. 4f), we then investigated the possibility that after stroke, SOM-negative interneurons had acquired SOM expression by performing co-staining with SOM and parvalbumin (PV), calretinin (Cr) and Choline acetyltransferase (ChAT). The results of these assessments were all negative (Fig. 4g–i).

To investigate the potential formation of new-born SOM+ interneurons, we treated mice with the thymidine analog BrdU for 2 weeks after stroke and assessed the number of double positive SOM+/BrdU+ cells 6 weeks thereafter. No co-staining was found between SOM and BrdU (Fig. 4j) indicating that these are not newborn cells related to stroke-induced neurogenesis.

Overall the results suggest that stroke increases the expression of SOM in interneurons, likely within SOM+ cells that weakly express SOM and are undetectable in absence of stroke injury.

## Discussion

The aim of this project, that is the extension of a previous study carried out by our group (Pintana et al. 2019), was to determine whether T2D affects the vulnerability of striatal SOM+ interneurons in the post-stroke recovery phase in the mouse. We demonstrated that stroke induces an increase in the number of SOM+ and NPY+ interneurons in the ipsilateral whole striatum of normal mice 6 weeks after stroke and that this effect is completely abolished by T2D. This T2D-mediated effect occurs specifically in the ischemic core but not in the peri-infarct stroke-damaged striatum. To generate these results, we setup a new computerized methodology that takes advantage of the complementary use of microscopy and an image-editing software. This method could reveal useful to also quantify other type of cells in striatum after ischemic damage.

SOM+ and NPY+ GABA-ergic interneurons have been shown to be involved in brain neuroplasticity (Tang et al. 2014). Specifically in relation to stroke, several groups have shown that after cerebral ischemia, SOM+/NPY+ expressing neurons in striatum are selectively spared (Uemura et al. 1990; Larsson et al. 2001), with an initial reduction in the first phases after surgery (4 h–1 day) and then return to baseline-like values 3–7 days after stroke (Grimaldi et al. 1990; Mallard et al. 1995) suggesting their potential role in post-stroke-related neuronal plasticity. Here, we confirm that these interneurons are spared in the first weeks after surgery (2 weeks) when also similar neurological recovery in SD and HFD-fed mice was observed (Pintana et al. 2019). However, we reported for the first time, that with longer time after stroke (6 weeks), the number of SOM and/or NPY expressing cell is further increased, exceeding also the baseline number. This stroke-related effect appears to occur in the ischemia injured striatum, and not because of a reduction of SOM+ cells in contralateral striatum, as no differences were detected in cell number between sham brains and contralateral hemispheres (Fig. S3). Remarkably, the number of these cells increased almost 40% in comparison to the heathy hemisphere 6 weeks after stroke indicating that this is not likely the result of an acute response to stroke. Whether the increase of SOM+ and/or NPY+ interneurons represents one of the mechanisms behind spontaneous neurological recovery after stroke is an attractive hypothesis to be proved in future studies. Although speculative, this could be the case, since the increase of SOM+ and/or NPY+ interneurons failed to occur in the same T2D mice where we also observed decreased neurological recovery (previously published in the first part of this study in Pintana et al. (2019). Indeed, decreased capacity to recover from stroke in T2D patients has long been established (Jorgensen et al. 1994; Baird et al. 2002; Megherbi et al. 2003) and represents an important clinical problem. Impaired stroke recovery in T2D depends, at least in part, on weakened neuroplasticity (Alia et al. 2017; Sweetnam et al. 2012; Tulsulkar et al. 2016; Dhungana et al. 2013), where interneurons play an important role (Calcagnotto 2016; Caillard et al. 2000). The identification of neuronal cell populations that could be involved in decreased recovery after stroke is very important in this respect and recent works in this field have suggested that PV+ interneurons could be involved in these effects (Park and Koh 2018; Pintana et al. 2019). Although speculative, the results of our study showing an increase of SOM+/NPY+ interneurons 6 weeks after stroke suggest that these cells could also be part of a “neuroplastic response” after stroke that is important for spontaneous stroke recovery and that could be hampered by T2D. This response could not be appreciated by counting SOM+/NPY+ in the whole striatum since the effect of T2D was specifically affecting the infarct but not the peri-infarct area. Although speculative, this observation of possible T2D-induced alteration of neuroplasticity involving SOM+/NPY+ interneurons could also open up for the possibility of developing new, targeted therapies to counteract negative effects of T2D on SOM+/NPY+ interneurons and facilitate stroke recovery.

Although no differences were found in average infarct volume between SD- and HFD-fed mice, as we demonstrated in these same mice in the first part of this study (Pintana et al. 2019), peri-infarct and infarct areas are variable between all mice, and this factor can affect the evaluation of raw or normalized cell number quantifications. Thereby a reasonable way to fairly compare cell number between animals was to calculate cell density (cell number divided by the area of infarct or peri-infarct area as described in “Materials and Methods” section). We then encountered two additional problems: (1) the tissue deformation 6 weeks after stroke (Ding et al. 2010; Li et al. 2014) and (2) the heterogeneous distribution of SOM+ cells in the striatum (Fig. 1). When it comes to the tissue deformation, literature clearly shows that after stroke the volume of infarct core varies considerably during time: in the first hours after stroke, an edema occurs, contributing to the swelling of infarct tissue that peaks at 1–2 days after surgery (Li et al. 2014). From this time point, the edema starts to regress and after 1–2 weeks the removal of dead tissue by macrophages and microglia results in the infarct area being gradually replaced by a scar tissue that leads to a reduction of the infarct volume (Macrae 2011). This time-dependent volume change (swelling/shrinking) after stroke, leads to the necessity to find corrections for morphological evaluations of stroke outcome; as an example, infarct volume can be assessed indirectly as the volume of the undamaged portion of the ipsilateral hemisphere subtracted from the volume of contralateral hemisphere and this method is based on the assumption that tissue volume changes almost exclusively within the infarct core (Macrae 2011). Concerning the evaluation of cell number in the infarct core (and comparison to contralateral hemisphere), one of the used techniques to overcome the problem of volume variations is to identify a region of interest (ROI) in the stroke affected area, and to select the symmetrical adjusted area in the contralateral hemisphere (Khodanovich et al. 2018). The evaluation of cell number/density in a small portion of infarct core (such as a selected ROI) could mask the variation of tissue swelling/shrinking, but, in case of not uniformly distributed cells as SOM+ interneurons, could also give a partial or even false result. Moreover, at longer time points (> 14 days) the deformation (shrinking) effect could be too evident to be masked by the isolation of a small ROI. Thereby, there is the need to find new methods to evaluate cell density in the striatum-damaged area discriminating between peri-infarct and infarct areas. From our previous data, we confirmed that only the infarct area is affected by tissue deformation, while the peri-infarct not-affected striatum does not change its volume during time (Fig. S2). The difference in the volume variation between the two areas is further endorsed by the universally accepted and above discussed method for infarct volume evaluation that assumes that the "not affected" brain area does not change its volume during time. As already discussed, this differential deformation of striatum must be taken in consideration in cell density quantifications, as a reduction of the area used to calculate cell density (that occurs only in infarct area) would create an artifact in the obtained values. This issue was overcome by adjusting cell density obtained to a deformation coefficient (*α*_s_), that represent the magnitude of infarct tissue deformation (shrinking in this case) for every single mouse. As the peri-infarct area is not affected by deformation (Fig. S2), the *α*_s_ obtained would have been equal to 1, and thus cell density in peri-infarct area did not need to be adjusted.

When it comes to the non-homogeneous distribution of SOM+ cell in the striatum, if cell density is evaluated by discriminating between different areas of the striatum, even the smallest difference in cell distribution should affect the reliability of the obtained results. As striatal SOM+ cells concentrate mainly nearby the lateral ventricle, that is the area usually less affected in focal stroke models, comparing the density of SOM+ cells in ipsilateral peri-infarct area to the density of the whole contralateral striatum, would result in a false increase of SOM+ cells. The striatal area nearby the lateral ventricle is, indeed, enriched of SOM+ cells in comparison to the whole striatum, even in physiological conditions. Thus, to obtain reliable results, peri-infarct and infarct cell density in ipsilateral striatum should be compared to the same corresponding area in the contralateral hemisphere. To identify accurately the corresponding peri-infarct and infarct areas in the contralateral hemisphere, we took advantage of a digital image software (Adobe Photoshop CC 2019, see “Materials and Methods” section for details).

In summary, the use the new method gave us the opportunity to evaluate SOM+ cell density in a precise and reproducible way, discriminating between peri-infarct and infarct areas and, in this way, to unmask an effect of T2D on SOM+ cells in the ischemic core that was otherwise undetectable by analyzing the whole striatum.

The last objective of this study was to understand the origin of these "new" detected SOM+ cells 6 weeks after stroke. For this reason, several co-immunostaining experiments with SOM were performed. First, we wanted to confirm the neuronal nature of these cells. From our data, 100% of SOM+ cells co-stained with NeuN, a marker of adult neurons, confirming that they are neurons. No co-staining was found between SOM and spiny neurons (detected by DARP 32) or other interneuron populations (PV+, Cr+, ChAT+) suggesting the unlikely stroke-induced expression of SOM in other neuronal population. No co-staining was detected between SOM and BrdU either, indicating that the increase of SOM+ cell number is not related to new-born cells/neurogenesis. However, as BrdU was injected only in the first 2 weeks after stroke, we cannot rule out (although unlikely) that the recorded increase of SOM+ cells at 6 weeks after stroke could be the result of a late (occurring after 2 weeks) neurogenesis process. Finally, we ruled out the potential expression of SOM in microglia, astroglia and oligodendrocytes. Based on these results, we speculate that the increase of SOM+/NPY+ neurons 6 weeks after stroke is the result of increased expression of these peptides in SOM+/NPY+ interneurons that, in normal uninjured conditions, do not produce levels of SOM or NPY detectable with standard immunohistological methods. However, to prove this hypothesis, the stroke-induced changes in expression of these peptides will need verification using more suitable methods, such as western blot analyses or rt-PCR. Unfortunately, in this study the brains were PFA-fixed, which prevented us from conducing such analyses, lack of which is a weakness of this study.

In conclusion we show that stroke increases the number of SOM+/NPY+ interneurons in the whole striatum during the recovery phase, likely in interneurons expressing SOM and/or NPY under undetectable levels in the uninjured brain. T2D completely abolished this effect in the ischemic core. The physiological meaning of this regulation is to date unknown but should be investigated by future studies aimed at identifying cellular targets to improve stroke recovery. We also show the development of a cellular counting system that allows precise, reliable and reproducible quantifications of potentially any cellular type in the stroke-damaged brain allowing to discriminate between infarct core and peri-infarct area and that takes in consideration tissue deformation and the not uniform distribution of cells in the considered brain area. The application of this method is simple and should bear great potential to quantify virtually any type of cells in the stroke damaged brain.

## Electronic supplementary material

Below is the link to the electronic supplementary material.

Supplementary file1 (DOCX 144 kb)

## Data Availability

All data are available upon request.
