# Supplementary material for: The Stroke-Induced Increase of Somatostatin-Expressing Neurons is Inhibited by Diabetes: A Potential Mechanism at the Basis of Impaired Stroke Recovery
Source: Cell Mol Neurobiol. 2020 May 23;41(3):591–603. doi: 10.1007/s10571-020-00874-7 (PMC7921043; doi:10.1007/s10571-020-00874-7)
Supplement: Supplementary file 1 — Supplementary file1 (DOCX 144 kb) [file 10571_2020_874_MOESM1_ESM.docx]

**The stroke-induced increase of somatostatin-expressing neurons is inhibited by diabetes: a potential mechanism at the basis of impaired stroke recovery**

Fausto Chiazza*^1,2^, Hiranya Pintana^1^, Grazyna Lietzau^1^, Thomas Nyström^1^, Cesare Patrone*^1^, Vladimer Darsalia*^1^

^1^Department of Clinical Science and Education, Södersjukhuset, Internal Medicine, Karolinska Institutet, Stockholm, Sweden

^2^Department of Pharmaceutical Sciences, Università degli Studi del Piemonte Orientale, Novara, Italy

Fausto Chiazza (Ph.D.) E-mail fausto.chiazza@uniupo.it

Hiranya Pintana (Ph.D.) E-mail [hiranya.pintana@ki.se](mailto:hiranya.pintana@ki.se)

Grazyna Lietzau (Ph.D.) E-mail [grazyna.lietzau@ki.se](mailto:grazyna.lietzau@ki.se)

Thomas Nyström (Ph.D., M.D.) E mail [thomas.nystrom@ki.se](mailto:thomas.nystrom@ki.se)

Cesare Patrone (Ph.D.) E-mail [cesare.patrone@ki.se](mailto:cesare.patrone@ki.se)

Vladimer Darsalia (Ph.D.) E-mail [vladimer.darsalia@ki.se](mailto:vladimer.darsalia@ki.se)

**CORRESPONDING AUTHORS:**

*Cesare Patrone (Ph.D.), Karolinska Institutet, Department of Clinical Science and Education, Södersjukhuset, Stockholm, Sweden. Phone: +46 (8) 6165084 Fax: +46 (8) 6162933. ORCID: [0000-0003-0470-4606](javascript:popup_orcidDetail('https://orcid.org','0000-0003-0470-4606');)

*Vladimer Darsalia (Ph.D.), Karolinska Institutet, Department of Clinical Science and Education, Södersjukhuset, Stockholm, Sweden. Phone: +46 (8) 6165084 Fax: +46 (8) 6162933. ORCID: 0000-0002-6693-934X

*Fausto Chiazza (Ph.D), Department of Pharmaceutical Sciences, Università degli Studi del Piemonte Orientale, Novara, Italy. Phone: +39 0321 375 829 ORCID: 0000-0001-7273-0051


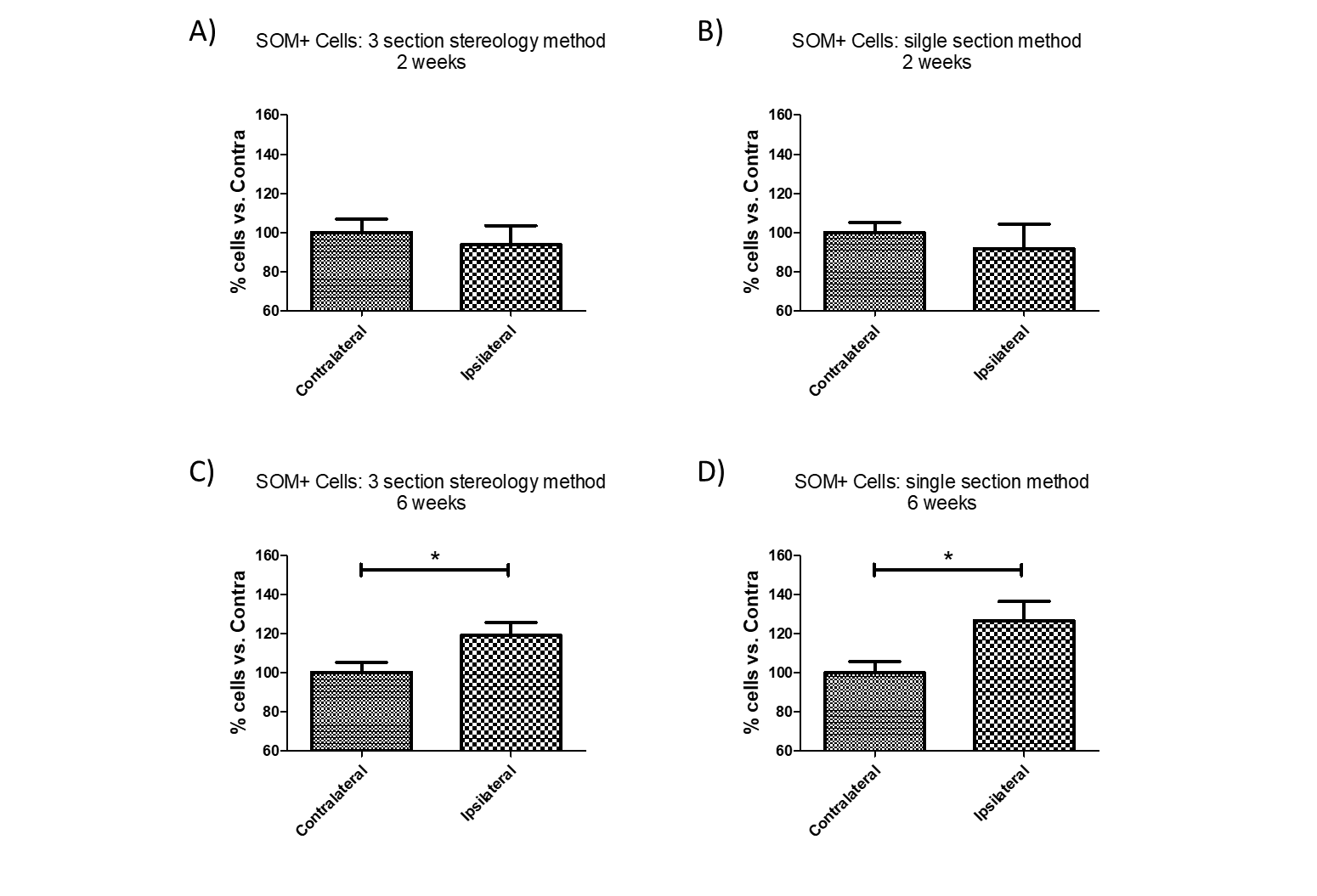
**Supplementary Fig. S1.** ***Comparison between 2 counting methods***

SOM positive cells number was evaluated at 2 and 6 weeks after stroke in SD fed animals by counting with stereology technique three consecutive brain sections (A and C, Method 1) or by counting all positive cells in the median of the three sections used for Method 1 (C and D, Method 2). Cell number was then normalized for the mean of respective contralateral cell number (5-6 animals per group). Unpaired t test with Welch’s correction, means ± SEM *p<0,05. No differences between the two counting methods were observed

**
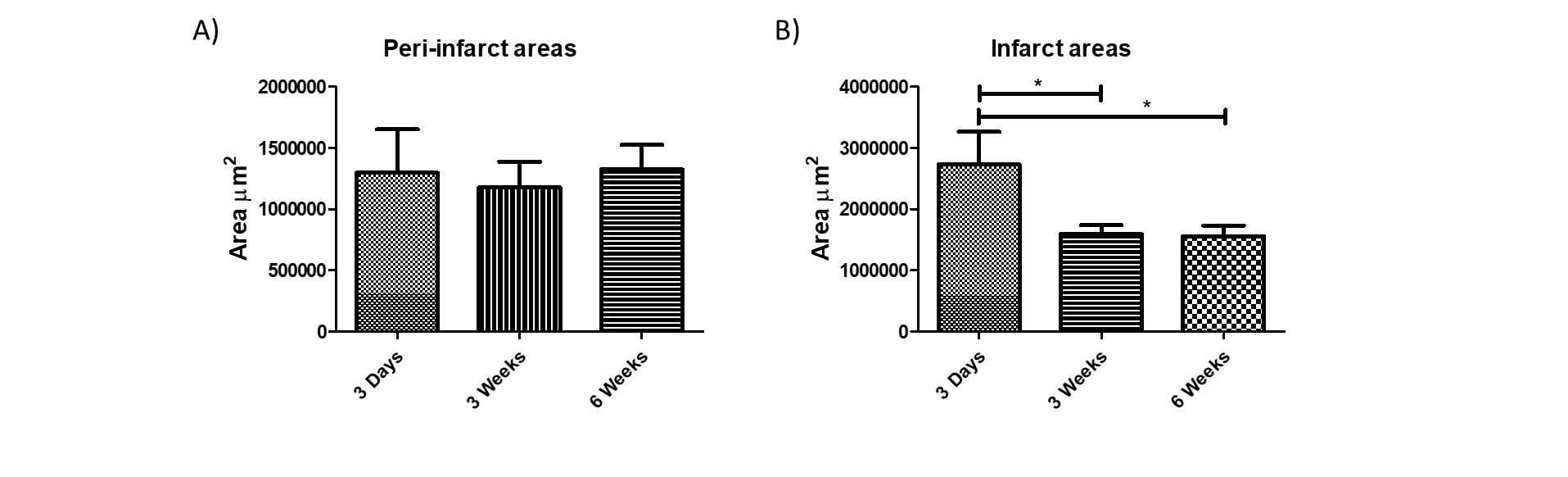
**

**Supplementary Fig. S2. *Comparison between peri-infarct and infarct areas at different time points***

The figure depicts the evaluation of Peri-Infarct (A) and Infarct (B) areas in a single section at different time-points (3 days, 3 weeks and 6 weeks after stroke, 5-10 animals per group). One-way ANOVA test with Bonferroni posttest, means ± SEM. *p<0,05. No differences between Peri-Infarct areas were detected, while an evident time-dependent infarct area shrinking can be observed

**Supplementary Fig.S3: *Evaluation of SOM+ cells in sham and contralateral striata of animals fed a SD or an HFD***

SOM positive cells number was evaluated at 6 weeks after stroke in SD and HFD fed animals by all positive cells in a single section (4-8 animals per group). One-way ANOVA test with Bonferroni posttest, means ± SEM. No differences in the total number of cells between Sham and Contralateral or between the 2 dietary regimes were observed.

**Extended statistics report**

| Fig 2A |  |  |  |  |  |
| --- | --- | --- | --- | --- | --- |
|  | **Bonferroni's multiple comparisons test** | **Mean Diff,** | **95,00% CI of diff,** | **Significant?** | **Summary** |
|  | Contralateral vs. Ipsilateral SD | 8,3 | -22,78 to 39,38 | No | ns |
|  | Contralateral vs. Ipsilateral HFD | 18,3 | -12,78 to 49,38 | No | ns |
|  | Ipsilateral SD vs. Ipsilateral HFD | 10 | -25,89 to 45,89 | No | ns |
|  |  |  |  |  |  |
| Fig 2B |  |  |  |  |  |
|  | **Bonferroni's multiple comparisons test** | **Mean Diff,** | **95,00% CI of diff,** | **Significant?** | **Summary** |
|  | Contralateral vs. Ipsilateral SD | -8,714 | -33,74 to 16,31 | No | ns |
|  | Contralateral vs. Ipsilateral HFD | 18,8 | -6,223 to 43,83 | No | ns |
|  | Ipsilateral SD vs. Ipsilateral HFD | 27,52 | -1,381 to 56,42 | No | ns |
|  |  |  |  |  |  |
| Fig 2C |  |  |  |  |  |
|  | **Bonferroni's multiple comparisons test** | **Mean Diff,** | **95,00% CI of diff,** | **Significant?** | **Summary** |
|  | Contralateral vs. Ipsilateral SD | -15,81 | -42,98 to 11,35 | No | ns |
|  | Contralateral vs. Ipsilateral HFD | 14,06 | -13,11 to 41,22 | No | ns |
|  | Ipsilateral SD vs. Ipsilateral HFD | 29,87 | -1,498 to 61,23 | No | ns |
|  |  |  |  |  |  |
| Fig 2D |  |  |  |  |  |
|  | **Bonferroni's multiple comparisons test** | **Mean Diff,** | **95,00% CI of diff,** | **Significant?** | **Summary** |
|  | Contralateral vs. Ipsilateral SD | -30,64 | -49,53 to -11,75 | Yes | *** |
|  | Contralateral vs. Ipsilateral HFD | -11,21 | -33,44 to 11,03 | No | ns |
|  | Ipsilateral SD vs. Ipsilateral HFD | 19,43 | -4,616 to 43,48 | No | ns |
|  |  |  |  |  |  |
| Fig 2E |  |  |  |  |  |
|  | **Bonferroni's multiple comparisons test** | **Mean Diff,** | **95,00% CI of diff,** | **Significant?** | **Summary** |
|  | Contralateral vs. Ipsilateral SD | -22,53 | -42,35 to -2,703 | Yes | * |
|  | Contralateral vs. Ipsilateral HFD | -12,86 | -34,91 to 9,189 | No | ns |
|  | Ipsilateral SD vs. Ipsilateral HFD | 9,664 | -14,65 to 33,97 | No | ns |
|  |  |  |  |  |  |
| Fig 2F |  |  |  |  |  |
|  | **Bonferroni's multiple comparisons test** | **Mean Diff,** | **95,00% CI of diff,** | **Significant?** | **Summary** |
|  | Contralateral vs. Ipsilateral SD | -32,28 | -54,30 to -10,27 | Yes | ** |
|  | Contralateral vs. Ipsilateral HFD | -22,57 | -47,06 to 1,924 | No | ns |
|  | Ipsilateral SD vs. Ipsilateral HFD | 9,717 | -17,28 to 36,72 | No | ns |

| Fig 3B |  |  |
| --- | --- | --- |
|  | **Unpaired t test with Welch's correction** |  |
|  | P value | 0,039 |
|  | P value summary | * |
|  | Significantly different (P < 0.05)? | Yes |
|  | One- or two-tailed P value? | Two-tailed |
|  | Welch-corrected t, df | t=2,315, df=12,10 |
|  |  |  |
|  | How big is the difference? |  |
|  | Mean of column A | 42,45 |
|  | Mean of column B | 61,53 |
|  | Difference between means (A - B) ± SEM | -19,08 ± 8,243 |
|  | 95% confidence interval | -37,02 to -1,137 |
|  | R squared (eta squared) | 0,3068 |
|  |  |  |
| Fig. 3C |  |  |
|  | **Unpaired t test with Welch's correction** |  |
|  | P value | 0,028 |
|  | P value summary | * |
|  | Significantly different (P < 0.05)? | Yes |
|  | One- or two-tailed P value? | Two-tailed |
|  | Welch-corrected t, df | t=2,393, df=17,70 |
|  |  |  |
|  | How big is the difference? |  |
|  | Mean of column A | 23,45 |
|  | Mean of column B | 31,24 |
|  | Difference between means (B - A) ± SEM | 7,796 ± 3,258 |
|  | 95% confidence interval | 0,9433 to 14,65 |
|  | R squared (eta squared) | 0,2445 |
|  |  |  |
| Fig 3D |  |  |
|  | **Unpaired t test with Welch's correction** |  |
|  | P value | 0,0202 |
|  | P value summary | * |
|  | Significantly different (P < 0.05)? | Yes |
|  | One- or two-tailed P value? | Two-tailed |
|  | Welch-corrected t, df | t=2,776, df=9,691 |
|  |  |  |
|  | How big is the difference? |  |
|  | Mean of column A | 40 |
|  | Mean of column B | 51,95 |
|  | Difference between means (B - A) ± SEM | 11,94 ± 4,303 |
|  | 95% confidence interval | 2,314 to 21,57 |
|  | R squared (eta squared) | 0,4429 |
|  |  |  |
| Fig 3E |  |  |
|  | **Unpaired t test with Welch's correction** |  |
|  | P value | 0,5622 |
|  | P value summary | ns |
|  | Significantly different (P < 0.05)? | No |
|  | One- or two-tailed P value? | Two-tailed |
|  | Welch-corrected t, df | t=0,6034, df=8,391 |
|  |  |  |
|  | How big is the difference? |  |
|  | Mean of column A | 26,96 |
|  | Mean of column B | 28,24 |
|  | Difference between means (B - A) ± SEM | 1,283 ± 2,127 |
|  | 95% confidence interval | -3,582 to 6,148 |
|  | R squared (eta squared) | 0,04159 |
|  |  |  |
| Fig 3F |  |  |
|  | Column A | %increase Peri-Infarct Area SD |
|  | vs. | vs, |
|  | Column C | % increase peri-infarct area HFD |
|  |  |  |
|  | **Unpaired t test with Welch's correction** |  |
|  | P value | 0,5434 |
|  | P value summary | ns |
|  | Significantly different (P < 0.05)? | No |
|  | One- or two-tailed P value? | Two-tailed |
|  | Welch-corrected t, df | t=0,6303, df=9,490 |
|  |  |  |
|  | How big is the difference? |  |
|  | Mean of column A | 141,7 |
|  | Mean of column C | 129,5 |
|  | Difference between means (A - C) ± SEM | 12,15 ± 19,27 |
|  | 95% confidence interval | -31,11 to 55,40 |
|  | R squared (eta squared) | 0,04018 |
|  |  |  |
|  |  |  |
|  | Column B | %increase Infarct Area SD |
|  | vs. | vs, |
|  | Column D | % increase infarct area HFD |
|  |  |  |
|  | **Unpaired t test with Welch's correction** |  |
|  | P value | 0,0209 |
|  | P value summary | * |
|  | Significantly different (P < 0.05)? | Yes |
|  | One- or two-tailed P value? | Two-tailed |
|  | Welch-corrected t, df | t=2,583, df=14,80 |
|  |  |  |
|  | How big is the difference? |  |
|  | Mean of column B | 135,6 |
|  | Mean of column D | 98,89 |
|  | Difference between means (B - D) ± SEM | 36,71 ± 14,21 |
|  | 95% confidence interval | 6,389 to 67,04 |
|  | R squared (eta squared) | 0,3108 |

| Fig S1A | |  |
| --- | --- | --- |
|  | **Unpaired t test with Welch's correction** |  |
|  | P value | 0,6102 |
|  | P value summary | ns |
|  | Significantly different (P < 0.05)? | No |
|  | One- or two-tailed P value? | Two-tailed |
|  | Welch-corrected t, df | t=0,5334, df=7,040 |
|  |  |  |
|  | How big is the difference? |  |
|  | Mean of column A | 100 |
|  | Mean of column B | 93,6 |
|  | Difference between means (B - A) ± SEM | -6,400 ± 12,00 |
|  | 95% confidence interval | -34,74 to 21,94 |
|  | R squared (eta squared) | 0,03885 |
|  |  |  |
| Fig S1B | |  |
|  | **Unpaired t test with Welch's correction** |  |
|  | P value | 0,5746 |
|  | P value summary | ns |
|  | Significantly different (P < 0.05)? | No |
|  | One- or two-tailed P value? | Two-tailed |
|  | Welch-corrected t, df | t=0,5977, df=5,311 |
|  |  |  |
|  | How big is the difference? |  |
|  | Mean of column A | 99,8 |
|  | Mean of column B | 91,6 |
|  | Difference between means (A - B) ± SEM | 8,200 ± 13,72 |
|  | 95% confidence interval | -26,45 to 42,85 |
|  | R squared (eta squared) | 0,06303 |
|  |  |  |
| Fig S1C | |  |
|  | **Unpaired t test with Welch's correction** |  |
|  | P value | 0,0457 |
|  | P value summary | * |
|  | Significantly different (P < 0.05)? | Yes |
|  | One- or two-tailed P value? | Two-tailed |
|  | Welch-corrected t, df | t=2,301, df=9,411 |
|  |  |  |
|  | How big is the difference? |  |
|  | Mean of column A | 100,2 |
|  | Mean of column B | 119,2 |
|  | Difference between means (A - B) ± SEM | -19,00 ± 8,258 |
|  | 95% confidence interval | -37,56 to -0,4434 |
|  | R squared (eta squared) | 0,36 |
|  |  |  |
| Fig S1D | |  |
|  | **Unpaired t test with Welch's correction** |  |
|  | P value | 0,0498 |
|  | P value summary | * |
|  | Significantly different (P < 0.05)? | Yes |
|  | One- or two-tailed P value? | Two-tailed |
|  | Welch-corrected t, df | t=2,323, df=7,720 |
|  |  |  |
|  | How big is the difference? |  |
|  | Mean of column A | 99,83 |
|  | Mean of column B | 128,7 |
|  | Difference between means (B - A) ± SEM | 28,83 ± 12,41 |
|  | 95% confidence interval | 0,03469 to 57,63 |
|  | R squared (eta squared) | 0,4115 |

| Fig. S2A | |  |  |  |  |
| --- | --- | --- | --- | --- | --- |
|  | **Bonferroni's multiple comparisons test** | **Mean Diff,** | **95,00% CI of diff,** | **Significant?** | **Summary** |
|  | 3 Days vs. 3 Weeks | 119402 | -880402 to 1119207 | No | ns |
|  | 3 Days vs. 6 Weeks | -27439 | -1132764 to 1077886 | No | ns |
|  | 3 Weeks vs. 6 Weeks | -146841 | -1015898 to 722216 | No | ns |
|  |  |  |  |  |  |
| Fig. 2SB | |  |  |  |  |
|  | **Bonferroni's multiple comparisons test** | **Mean Diff,** | **95,00% CI of diff,** | **Significant?** | **Summary** |
|  | 3 Days vs. 3 Weeks | 1137597 | 186667 to 2088527 | Yes | * |
|  | 3 Days vs. 6 Weeks | 1170932 | 119639 to 2222225 | Yes | * |
|  | 3 Weeks vs. 6 Weeks | 33335 | -793239 to 859909 | No | ns |
|  |  |  |  |  |  |
| Fig S3 |  |  |  |  |  |
|  | **Bonferroni's multiple comparisons test** | **Mean Diff,** | **95,00% CI of diff,** | **Significant?** | **Summary** |
|  | Sham SD vs. Contralateral SD | 0,4 | -23,49 to 24,29 | No | ns |
|  | Sham SD vs. Sham HFD | 5,25 | -19,93 to 30,43 | No | ns |
|  | Sham SD vs. Contralateral HFD | -6,429 | -32,49 to 19,64 | No | ns |
|  | Contralateral SD vs. Sham HFD | 4,85 | -19,04 to 28,74 | No | ns |
|  | Contralateral SD vs. Contralateral HFD | -6,829 | -31,65 to 17,99 | No | ns |
|  | Sham HFD vs. Contralateral HFD | -11,68 | -37,74 to 14,39 | No | ns |
